# Adverse Cell Culture Conditions Mimicking the Tumor Microenvironment Upregulate ABCG2 to Mediate Multidrug Resistance and a More Malignant Phenotype

**DOI:** 10.5402/2012/746025

**Published:** 2012-06-14

**Authors:** Grace M. Y. Cheng, Kenneth K. W. To

**Affiliations:** School of Pharmacy, The Chinese University of Hong Kong, Hong Kong

## Abstract

ABCG2 is an efflux transporter commonly found to overexpress in multidrug resistant (MDR) cancer cells. It is also believed to be a survival factor for cancer stem cells to drive tumor growth. Tumor microenvironment represents an attractive new drug target because it allows complex interaction between a tumor and its surrounding normal cells, molecules, and blood vessels, which all participate in tumor progression. Hypoxia, glucose deprivation and acidosis are the hallmarks of tumor microenvironment. This study investigated the upregulation of ABCG2 by these adverse growth conditions within the tumor microenvironment. Reporter gene assay revealed that a region within the *ABCG2* promoter close to the reported HIF-1**α** response element is responsible for ABCG2 upregulation. Increased ABCG2 efflux activity was observed under the same conditions, subsequently leading to reduced response to ABCG2 substrate anticancer drug. Importantly, glucose deprivation and hypoxia were also found to enhance the resistance level of ABCG2-overexpressing resistant cells with pre-existing genetic and epigenetic MDR mechanisms. Hypoxia was further demonstrated to cause a more malignant anchorage-independent growth phenotype in the resistant cells, which can be abolished by knocking down ABCG2. A better understanding of ABCG2 regulation by the tumor microenvironment may help design novel strategies to improve treatment outcome.

## 1. Introduction

Multidrug resistance (MDR) remains a major unresolved obstacle to successful cancer chemotherapy. It is usually associated with an increased efflux of cytotoxic drugs by ATP-binding cassette (ABC) transporters including ABCG2. ABC transporters are energy-dependent transporters that normally function in the detoxification and protection of normal cells from xenobiotics. Overexpression of ABCG2 in cancer cell lines *in vitro* has been shown to confer MDR to a variety of anticancer drugs including mitoxantrone, irinotecan, methotrexate, flavopiridol, and anthracyclines [1]. Moreover, growing evidence suggests that ABCG2 underlies the MDR of clinical samples from different cancers [2]. ABCG2 also plays a critical role in hypoxic defense mechanisms within the tumor microenvironment [3]. It may also contribute to the maintenance of stem or progenitor cells as a survival factor, ultimately driving tumor growth [4]. Identification of factors that can influence ABCG2 expression and activity might lead to the development of new strategies to modulate ABCG2-mediated drug transport clinically. 

Cancer cells do not exist in isolation. They can be thought of as inhabiting within a complex milieu of normal cells, blood vessels, endogenous small molecules, and secreted factors, which collectively comprise the tumor microenvironment. Recent advances have indicated that the tumor microenvironment is critically important for cancer initiation, progression, metastasis, and drug resistance, thus providing opportunities for therapeutic intervention. 

This study aims to evaluate the regulation of ABCG2 in response to a few characteristic growth conditions within the tumor microenvironment, including hypoxia, glucose deprivation, and low pH. Cancer cells are often confronted with a remarkable reduction in oxygen supply inside solid tumors, leading to intratumoral hypoxia. Hypoxia-inducible factor (HIF-1*α*), the master regulator of hypoxic response, is accumulated in order to adapt to the adverse growth condition. The production of vascular endothelial growth factor (VEGF) and other hypoxia-induced angiogenic cytokines to promote increase tissue vascularization, and the metabolic switch from oxidative to glycolytic metabolism represent the two major adaptive responses to enhance cell survival under tumor hypoxic condition. Glucose depletion is often observed in malignant tumors due to insufficient blood supply in the core of solid tumors [5] and increased rate of glycolysis [6]. On the other hand, the production of lactic acid under anaerobic conditions and the hydrolysis of ATP in an energy-deficient environment contribute to the acidic microenvironment that has been shown in many types of tumor (Warburg's effect [7]). While the effect of tumor microenvironment on oncogenes and tumor suppressor genes have been extensively studied recently [8], less is known about how this may modulate the xenobiotic transporters thus mediating multidrug resistance. A better understanding of this complicated interaction may allow us to design novel therapeutic strategies to improve treatment outcome in chemotherapy. 

## 2. Materials and Methods

### 2.1. Chemicals

Cisplatin was obtained from Strem Chemicals (Newburyport, MA). Mitoxantrone and 2-deoxyglucose (DG) were purchased from Sigma Chemical (St. Louis, MO). Pheophorbide A (PhA) and Fumitremorgin C (FTC) were kind gifts obtained from Dr. Susan Bates (National Cancer Institute, NIH, Bethesda, MD, USA). 

### 2.2. Cell Culture and Growth Conditions

Human colon carcinoma HCT-116, and S1 and its ABCG2-overexpressing resistant S1M1-80 cell lines were kindly provided by Dr. Susan Bates (National Cancer Institute, NIH, USA). S1 and S1M1-80 have been described previously [9]. The resistant S1M1-80 subline used in this study was derived from the S1 clone of the LS174 human colon cancer cell line by prolonged selection in mitoxantrone, which was routinely maintained at 80 *μ*M mitoxantrone. There is no amplification of the *ABCG2* gene in S1M1-80. The ABCG2-mediated drug resistance in S1M1-80 is believed to be contributed by a balanced t(4,7) translocation downstream of the *ABCG2* gene [10] and the escape from miR-519c repression by a shortening of its 3′untranslated region [11]. At the time of investigation, it has been allowed to grow in drug-free medium for at least 3 weeks. The cell lines were maintained in RPMI 1640 medium supplemented with 10% fetal bovine serum, 100 units/mL streptomycin sulfate, and 100 units/mL penicillin G sulfate, and incubated at 37°C in 5% CO_2_.

Hypoxic incubation was accomplished by using the Bactron Anaerobe System (Sheldon Manufacturing Inc., Cornelius, OR, USA) as previously described [12]. Prior to hypoxic incubation, the culture was replenished with fresh complete medium and the cells were then subjected to 3 cycles of evacuation refilling of anaerobic gas (90% nitrogen and 10% CO_2_). The hypoxic environment was proved to be radiobiologic hypoxic (OER = 3.0, data not shown). 

To mimic glucose depletion, cells were cultured in glucose-free RPMI 1640 medium for 16 h or in regular medium but in the presence of 20 mM 2-deoxyglucose (2-DG) for 16 h. Under these conditions, no significant cell death was observed. 

To test the effect of acid, the glucose-containing culture medium was titrated with 0.1 M HCl to pH 5. In order to ensure that any observed change in the transporter expression was not attributed by a change in medium osmolality, control cells were also grown in glucose-containing RPMI-1640 with an added volume of distilled water to achieve an osmolality equivalent to the acid-treated medium (~20% v/v of added water). 

### 2.3. Reverse Transcription and Quantitative Real-Time PCR. 

Total RNA was isolated using the Trizol regent (Invitrogen, Carlsbad, CA). RNA (1 *μ*g) was reverse transcribed using the Transcriptor High Fidelity cDNA Synthesis Kit (Roche Applied Science, Indianapolis, IN). Quantitative real-time PCR was performed to quantify the change of ABCG2 or PGK1 transcript expressions using the KAPA SYBR FAST qPCR Kit (KapaBiosystems, Woburn, MA) in a LightCycler 480 Instrument I (Roche Applied Science, Indianapolis, IN). The human GAPDH RNA was amplified in parallel as the internal control. The specific primers used are as follows: ABCG2 (forward) 5′-TTTCCAAGCGTTCATTCAAAAA-3′ (reverse) 5′-TACGACTGTGACAATGATCTGAGC-3′; PGK1 (forward) 5′-TTTCTAACAAGCTGACGCTG-3′, (reverse) 5′-TTCTTCCTCCACATGAAAGC-3′; GAPDH (forward) 5′-AGCCACATCGCTCAGACAC-3′ (reverse) 5′-GTTCAAACTTCTGCTCCTGA-3′. PCRs were performed at 95°C for 5 min, followed by 50 cycles of 95°C for 10 s and 60°C for 10 s. Fluorescence signal was acquired at the end of the elongation step of every PCR cycle (72°C for 10 s) to monitor the increasing amount of amplified DNA. ΔCt was calculated by subtracting the Ct of GAPDH from the Ct of the transcript under investigation. ΔΔCt was then calculated by subtracting the ΔCt of the untreated cells (or parental cells) from the ΔCt of the treated cells (or resistant cells). Fold change of gene expression was calculated by the equation 2^−ΔΔCT^.

### 2.4. RNA Interference

A small interference hairpin-loop (sh) silencing vector against the human ABCG2 (pU6-ABCG2) and a negative control shRNA vector targeting firefly luciferase (pU6-Luc) were prepared as described in To et al. [13]. S1 and S1M1-80 cells were transfected with pU6-Luc or pU6-ABCG2 using Lipofectamine 2000 (Invitrogen, Grand Island, NY). Silencing efficiency was assessed by RT-PCR and immunoblot analysis. 

### 2.5. Luciferase Reporter Assay

A series of human *ABCG2* promoter constructs with progressive deletions at the 5′-end has been described previously [14]. The *ABCG2* promoter/firefly luciferase fusion genes (400 ng DNA) were transfected in HCT-116 or S1 cells on 24-well plates using Fugene 6 (Roche, Indianapolis, IN). The pGL3-Basic (promoterless) plasmid, encoding firefly luciferase (Promega, Madison, WI), was used to determine the basal levels. In each experiment, the phRG-Basic plasmid (100 ng), encoding Renilla luciferase (Promega), was cotransfected for normalization purposes. Luminescence was measured 48 h after transfection using the Dual-Luciferase Reporter Assay System (Promega) with the GloMax 20/20 luminometer (Promega). In experiments involving 2-DG or hypoxic treatment, the cells were treated at 24 h after transfection for 16 h. Reporter activity was normalized by calculating the ratio of firefly/renilla values. Results were expressed as mean ± SD of duplicate measurements from three independent transfections.

### 2.6. ABCG2 Transport Activity by Flow Cytometry

The ABCG2-mediated efflux activity in HCT-116, S1 and S1M1-80 cells with or without pretreatment with 2-DG or hypoxia were determined by flow cytometric assays as described previously [13]. Trypsinized cells was incubated in 1 *μ*M PhA with or without 10 *μ*M ABCG2-specific inhibitor FTC in complete medium (phenol red-free RPMI 1640 with 10 % FBS) at 37°C in 5% CO_2_ for 30 min. Subsequently, the cells were washed with cold complete medium and then incubated for 1 h at 37°C in PhA-free medium continuing with FTC to generate the FTC/efflux histogram, or without FTC to generate the efflux histogram. The FTC-inhibitable PhA efflux was determined as the difference in mean fluorescence intensity (ΔMFI) between the FTC/efflux and efflux histograms, which indicates the ABCG2-mediated transport activity. Cells were finally washed with cold Dulbecco's PBS and placed on ice in the dark until analysis by flow cytometry. To measure the cell surface ABCG2 expression, trypsinized cells were incubated in 2% bovine serum albumin/Dulbecco's PBS with either phycoerythrin-conjugated anti-ABCG2 antibody 5D3 (eBioscience, San Diego, CA) or phycoerythrin-conjugated mouse IgG2b negative control antibody (eBioscience) according to manufacturer's instructions for 30 min at room temperature. The cells were then washed with Dulbecco's PBS and subsequently analyzed. Surface expression of ABCG2 was calculated as the difference in mean channel numbers between the 5D3 antibody histogram and the negative control antibody histograms. 

Samples were analyzed on an LSRFortessa Cell Analyzer (BD Biosciences, San Jose, CA). Phycoerythrin fluorescence was detected with a 488 nm argon laser and a 585 nm bandpass-filter, whereas PhA fluorescence was detected with a 488 nm argon laser and a 670 nm bandpass filter. At least 10,000 events were collected for all flow cytometry studies. Cell debris was eliminated by gating on forward versus side scatter and dead cells were excluded based on propidium iodide staining. All assays were performed in three independent experiments. 

### 2.7. Growth Inhibition Assay

Growth inhibitory effect of mitoxantrone and cisplatin (representing a typical ABCG2 substrate and nonsubstrate, resp.), with or without the pretreatment of 2-DG or hypoxia, on HCT-116, S1 and S1M1-80 cells, were evaluated by the sulforhodamine B assay [15]. Cells were seeded into 96-well microtitre plates in 100 *μ*L at a plating density of 5,000 cells/well and allowed to incubate overnight. The cells were then exposed to 2-DG (20 mM) or hypoxia for 16 h, before being treated with mitoxantrone or cisplatin at a range of concentrations and allowed to incubate at 37°C in 5% CO_2_ for another 72 h. Each drug concentration was tested in quadruplicate and controls were tested in replicates of eight. Each experiment was carried out independently at least three times. To determine whether differences between IC_50_ values were significant, the Student's *t*-test was performed with *P* < 0.05 being considered significant. 

### 2.8. Soft Agar Colony Formation Assay

Soft agar plates were prepared in six-well plates with a bottom layer of 0.6% Noble agar in serum-free RPMI 1640. 1,000 cells, with or without a 24 h hypoxia treatment, were suspended in 0.3% Noble agar in RPMI 1640 supplemented with 10% fetal bovine serum and seeded onto the bottom layer. Plates were then incubated for 3 weeks in a 37°C incubator. The number of colonies was counted after staining with 0.05% crystal violet for 1 h. 

## 3. Results 

### 3.1. Glucose Depletion, Decreased Extracellular pH, and Hypoxia Upregulate ABCG2 Transcript Level

**Figure 1:**
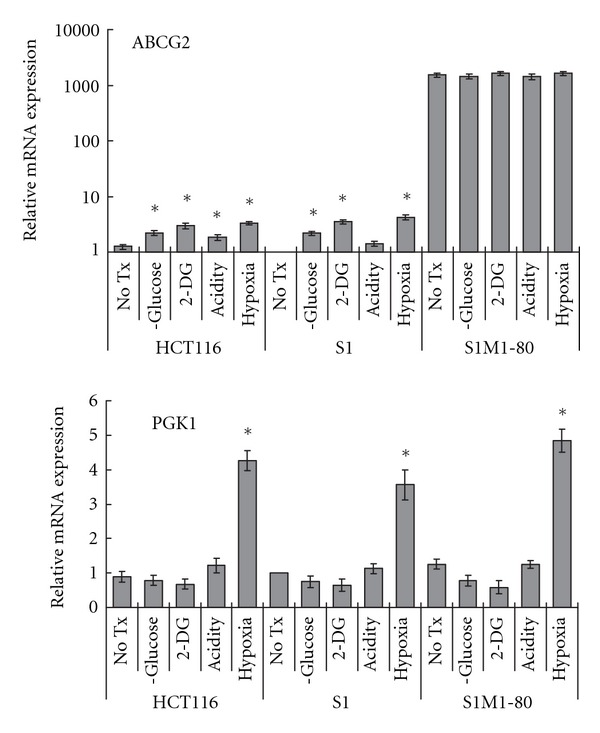
Quantitative real-time RT-PCR analysis showing the upregulation of ABCG2 in S1 and HCT-116 pretreated for 16 h with 2-DG (20 mM), low pH (pH 5) or hypoxia. 16 h treatment was selected because HIF-1*α* induction was found to reach the maximum after exposure to 16 h hypoxia treatment (data not shown). Expression of PGK1, a hypoxia response gene, was also measured to indicate the activation of the glycolytic pathway by hypoxia. mRNA expressions were normalized with GAPDH. Results for each gene are expressed relative to that in the untreated cells (2-DG = 2-deoxyglucose). Mean ± SD from three independent experiments is shown. The Student's *t*-test was used to compare the gene expression between the cells with or without the various treatment (*P < 0.05). ABCG2 mRNA expression was not affected significantly after the various treatments in S1M1-80 cells.

 The ABCG2 mRNA expression was evaluated in three human colon carcinoma cell lines (HCT-116, S1 and its resistant subline S1M1-80) after the pretreatment with glucose depletion, 2-DG, acidic pH and hypoxia. In HCT-116 and S1, a small but significant upregulation of ABCG2 (2 to 4-fold) was observed under most of these conditions (Figure 1, upper panel). Acidic pH did not significantly change ABCG2 level in S1 cells. Since the effect was more pronounced after 2-DG and hypoxia treatment, these conditions were selected for later functional and more detailed mechanistic studies. On the other hand, ABCG2 mRNA expression was not changed in the resistant S1M1-80 cells. The expression of PGK1, a hypoxia responsive gene, was also measured to verify the hypoxia treatment. Our data indicates that PGK1 was remarkably induced by the hypoxia treatment in all three cell lines (Figure 1, lower panel). The increase in ABCG2 mRNA after 2-DG or hypoxia treatment was blocked by actinomycin D (5 *μ*g/mL; data not shown), suggesting that these tumor microenvironment conditions regulate ABCG2 mRNA levels through an effect on ABCG2 transcription. 

### 3.2. A Cis-Element Close to the Reported HIF-1*α* Response Element at the *ABCG2* Promoter Is Required for the Upregulation of *ABCG2* after 2-DG and Hypoxia Treatment 

**Figure 2:**
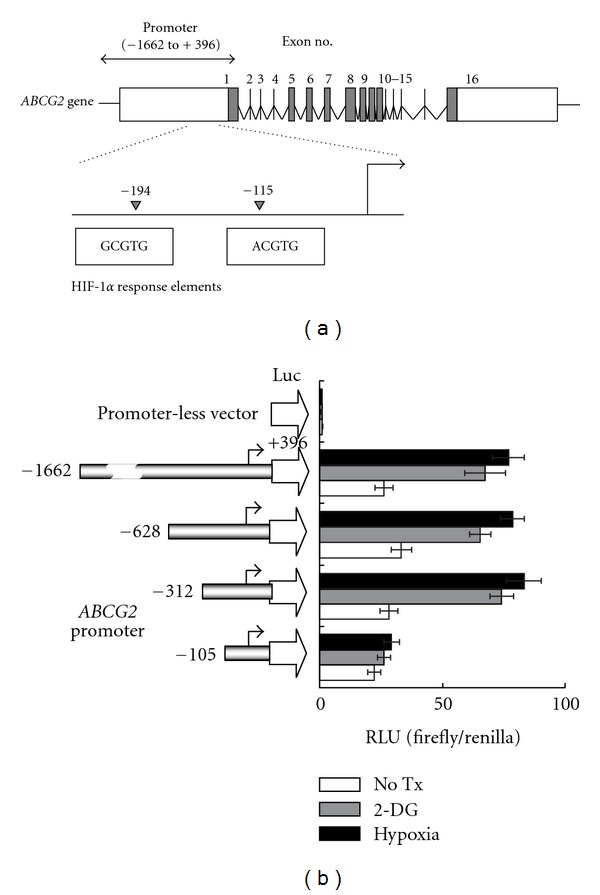
*ABCG2* promoter luciferase reporter gene assay showing that the promoter region harboring the HIF-1*α* response element is required for the activation of *ABCG2* in S1 human colon cancer cell line. Similar results were also obtained for HCT-116, which are shown in Supplementary Figure  1. (a) Schematic representations of the various 5′ deleted *ABCG2*-promoter constructs. The 5′-end of each of the constructs relative to the transcription start site (arrows) is indicated. The pGL3-basic (promoterless) vector, encoding firefly luciferase, was used to determine the basal levels. The last construct (−105/+396) does not contain the HIF-1*α* response element. (b) *ABCG2* reporter activity was measured in S1 cells transiently transfected with the various ABCG2 promoter constructs with or without 24 h pretreatment with 2-DG (20 mM) or hypoxia. The mean reporter activity ± SD (firefly/renilla luciferase units (RLU)) from three independent experiments is presented.

A series of 5′-deletion reporter gene constructs harboring the *ABCG2* promoter region with the 3′ end terminating at +396 bp were used to elucidate the promoter region responsible for glucose depletion or hypoxia-mediated ABCG2 upregulation (Figure 2(b)) [14]. After transiently transfected into HCT-116 and S1 cells, all the *ABCG2* promoter-luciferase constructs demonstrated good activities above the promoter-less pGL3—basic background (data for HCT-116 and S1 are shown in Figure 2(b) and Supplementary Figure  1 (available online at doi:10.5402/2012/746025), resp.). While reporter activity of the full length *ABCG2* promoter construct (−1662/+396) and the longer ones (−628 and −312/+396) were found to be activated in HCT-116 and S1 pretreated with 2-DG or hypoxia, the activation was abolished in the shortest construct (−105/+396). The data implies that the region between −312 and −105, harboring two reported HIF-1*α*  response elements [3] (Figure 2(a)), is responsible for the observed induction of ABCG2 transcription. 

### 3.3. 2-DG and Hypoxia Treatment Increased ABCG2-Mediated Drug Efflux and Reduced Cytotoxic Effects of ABCG2 Substrate Drugs

The upregulation of ABCG2 observed above suggested that all of the tumor microenvironment conditions investigated may confer a more drug-resistant phenotype. Next we sought to demonstrate the functional evidence with special regard to drug efflux activity and *in vitro* drug response. 

**Figure 3:**
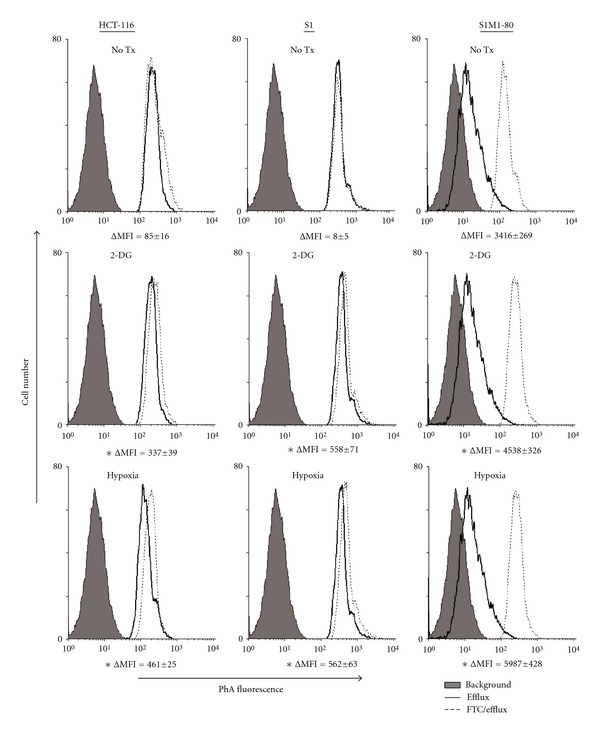
Drug efflux assay showing the upregulation of ABCG2-mediated drug efflux in HCT-116, S1 and S1M1-80 cells after a 16 h pretreatment with 2-DG (20 mM) or hypoxia. ΔMFI was determined as the difference in mean fluorescence intensity between the FTC/efflux and efflux histograms, which indicates the ABCG2-mediated transport activity. *P < 0.05, significantly different from the untreated HCT-116, S1, S1M1-80 cells, respectively.

By flow cytometric analysis, both 2-DG and hypoxia treatment led to a small but statistically significant elevation of ABCG2-mediated drug efflux (ΔMFI = signal between the efflux and FTC/efflux histograms) in all cell lines tested (Figure 3). Since the basal ABCG2 levels in S1 and HCT-116 cells are very low, we did not proceed to measure the protein expression either by Western blot or surface staining by flow cytometry. Nonetheless and more importantly, 2-DG and hypoxia were found to significantly decrease the cytotoxic effect of the ABCG2 substrate anticancer drug (mitoxantrone) (Table 1). On the other hand, the cytotoxicity of cisplatin was not affected, indicating the specific effect of these tumor microenvironment conditions on ABCG2-mediated MDR (Table 1). 

**Figure 4:**
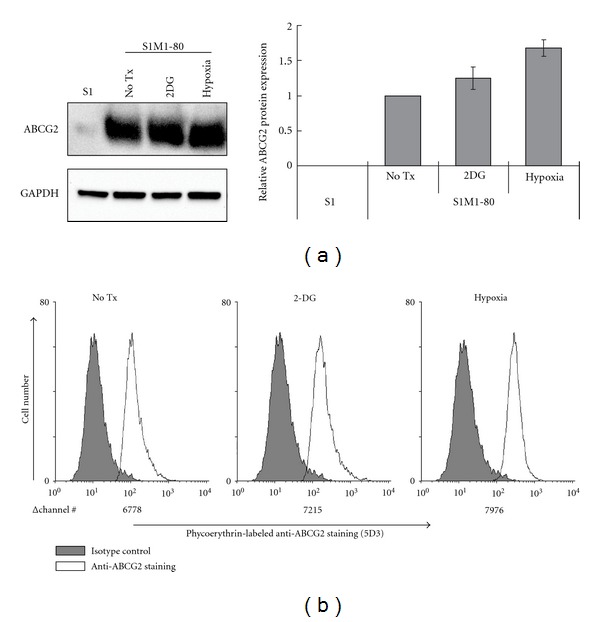
Western blot analysis and flow cytometric assay showing a slight elevation of ABCG2 surface expression by 2-DG and hypoxic treatment in S1M1-80 cells. (a) Left panel: Western blot analysis showing ABCG2 protein expression in whole cell lysate obtained from S1M1-80 cells after the indicated treatment; right panel: relative ABCG2 protein expression based on densitometric analysis of Western blot images. (b) Flow cytometry-based cell surface ABCG2 staining. S1M1-80 cells were exposed to 2-DG (20 mM) or hypoxia for 16 h, after which they were incubated with anti-ABCG2 antibody (5D3) (denoted by the solid black line) or a negative control antibody (denoted by the grey-filled histogram). Fluorescence was then determined by flow cytometry. Δchannel # represents the difference in fluorescence signal between the specific ABCG2 antibody staining and the isotype antibody control. Representative histograms from one of three independent experiments are shown.

In the ABCG2-overexpressing resistant S1M1-80 cells, Western blot and flow cytometry-based cell surface staining analysis were also performed because ABCG2 protein level is readily detectable. Interestingly, by both assays, ABCG2 protein expression was also found to be induced slightly after 2-DG and hypoxia treatment (Figures 4(a) and 4(b)). In Figure 4(b) (flow cytometry cell surface staining), the Δchannel # between the specific anti-ABCG2 staining and the isotype antibody control represents the cell surface ABCG2 protein expression. Importantly, this slight induction of ABCG2 protein by 2-DG and hypoxia was also found to decrease the cytotoxic effect of mitoxantrone (an ABCG2 substrate anticancer drug) (Table 1), probably through enhancing the ABCG2 efflux activity (Figure 3). In an attempt to further understand the induction of ABCG2 protein expression by the 2-DG and hypoxia treatment, cycloheximide chase assay was performed to evaluate the degradation rate of ABCG2 protein (data not shown). Unfortunately, ABCG2 protein was found to be fairly stable and no appreciable degradation was observed with or without the 2-DG or hypoxia treatment after up to 36 h. 

### 3.4. 2-DG and Hypoxia Enhanced the Anchorage Independent Cell Growth in Soft Agar Colony Formation Assay

**Figure 5:**
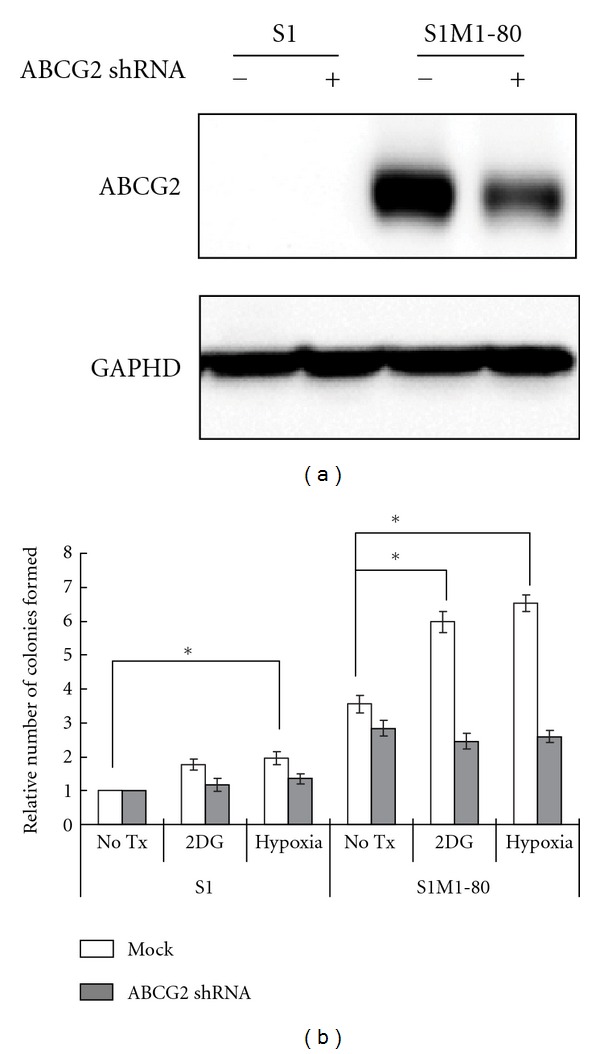
Soft agar colony formation assay showing the increased tendency of S1 or S1M1-80 cells to form anchorage-independent colonies after a 3-week incubation, which was abolished by knocking down ABCG2 by silencing vector. (a) Silencing efficacy of the ABCG2 shRNA as revealed by Western blot analysis. ABCG2 protein expression was decreased by ~50% in the shRNA-transfected S1M1-80 cells. (b) Relative no. of colonies formed in S1 or S1M1-80 cells after the indicated treatment. *P < 0.05, compared with the no-treatment group.

Anchorage-independent growth is one of the hallmarks of cell transformation, which is considered a reliable *in vitro* assay for detecting malignant transformation of cells. Since ABCG2 upregulation was observed after 2-DG and hypoxia treatment and that ABCG2-mediated MDR may lead to a more malignant phenotype, the colony formation capability of S1 and S1M180 cells with or without 2-DG and hypoxia treatment was assessed. In both cell lines, hypoxia was found to significantly increase the colony formation capability (Figure 5(b)). In S1M1-80 cells, 2-DG treatment was also found to enhance the tendency to form colony in a statistical significant manner. Interestingly, the colony formation capability could be abolished when ABCG2 was knocked down in the cells (Figures 5(a) and 5(b)). 

## 4. Discussion

The concept of tumor microenvironment is gaining a lot of attention in recent years because it allows complex interaction between a tumor and its surrounding normal cells, molecules, and blood vessels, which all participate in tumor progression [16]. A tumor can change its microenvironment and the microenvironment can affect the way the tumor grows and spreads. Hypoxia, glucose deprivation, and acidosis are generally considered as the hallmarks of the tumor microenvironment. 

While chemotherapy is the mainstay of treatment strategy for cancer, drug resistance is seriously hindering the clinical efficacy of most anticancer drugs. Solid tumors exhibit distinct structural abnormalities such as the leaky vasculature and thus have poor tissue perfusion, subsequently leading to *physiological* resistance to anticancer drugs. On the other hand, resistant cancer cells often express high levels of the multidrug resistance (MDR) transporters as their *biochemical* mechanism of drug resistance. The MDR transporters are efflux transporters on cell surface. They make use of the energy derived from ATP hydrolysis to regulate intracellular drug concentration, thereby determining cell sensitivity to chemotherapeutic agents. ABCG2 is one of these transporters, conferring MDR to a broad spectrum of antitumor agents. With high normal tissue expression in the brain endothelium, gastrointestinal tract, and placenta, ABCG2 is believed to be important in the protection from xenobiotics, regulating oral bioavailability, forming part of the blood-brain barrier, and the maternal-fetal barrier. Importantly, ABCG2 may also contribute to the maintenance of stem or progenitor cells as a survival factor. Among the various conditions within the tumor microenvironment, hypoxia has been demonstrated to upregulate ABCG2 in a mouse progenitor cell model [3]. This study attempted to understand the regulation of ABCG2 by the various adverse growth conditions within the tumor microenvironment in colon carcinoma cell lines. 

In fact, it has been postulated that the ATP-dependent drug efflux may be reduced under hypoxic condition. Cells are known to switch from oxidative to glycolytic metabolism under hypoxia and glycolytic pathway is less efficient in generating ATP [17]. To this end, it has been shown that a limitation in ATP supply (by sodium azide or 2-DG) could inhibit daunorubicin efflux in a non-Pgp-overexpressing resistant cell line [18]. Xu et al. demonstrated that the inhibition of mitochondrial respiration leads to an increase in the cytotoxic efficacy of various chemotherapeutic drugs as a result of a reduced P-gp activity which was attributed to a depletion of ATP [19]. On the other hand, it has been shown that acidosis (especially together with hypoxia) could increase P-gp-mediated drug efflux and lead to reduced cytotoxicity of chemotherapeutic drugs [20]. The discrepancy between the studies may be caused by the different cell lines used and different extent of hypoxia treatment. 

Our study focused on ABCG2, which is a more recently discovered MDR transporter. The data revealed that hypoxia, glucose deprivation, and acidosis can all upregulate ABCG2 in the two parental colon cancer cell lines (Figure 1) and apparently leading to multidrug resistance (Table 1). The upregulation is likely mediated at the transcription level because it can be abolished by pretreatment with actinomycin D, which inhibits RNA synthesis (data not shown). The effect on an ABCG2-overexpressing resistant S1M1-80 cell line was much less pronounced at the RNA level (Figure 1). However and interestingly, the resistance level was further enhanced in S1M1-80 cells after 2-DG and hypoxia treatment (Table 1). This effect may have been mediated at the protein level (see below). Since hypoxic condition will commit the cells to glycolytic metabolism, a downstream glycolytic gene (*PGK1*) was also evaluated in our study (Figure 1) as a surrogate for the hypoxic treatment and to demonstrate the functional linkage with glucose metabolism. As expected, PGK1 level was elevated remarkably after the hypoxia treatment. To evaluate the effect of glucose depletion, cells were grown either in glucose-free medium or in glucose-containing medium supplemented with 2-DG. 2-DG is a glucose analogue, which acts as a competitive inhibitor of glucose metabolism [21], where 2-DG competes with D-glucose to be transferred to the cells as a target for the hexokinase in the first step of the glycolytic pathway. Upon transport into the cells, glucose and 2-DG are phosphorylated to glucose-PO_4_ and 2-DG-PO_4_, respectively, by the hexokinase. However, unlike glucose-PO_4_, the 2-DG-PO_4_ cannot further be metabolized by the glucose phosphate isomerase (GPI). In addition, 2-DG could inhibit GPI [22], and then downregulate its downstream glycolysis-related genes (such as PGK1), which agree well with our findings (Figure 1). Regarding the effect of acidosis, the upregulation of ABCG2 was only statistically significant in HCT-116 cells. 

Since ABCG2 upregulation is more pronounced after 2-DG and hypoxia treatment, further mechanistic and functional studies were performed under these conditions. By reporter gene assay, the *ABCG2* promoter region (−312 to −105) close to the reported HIF-1*α* response element was found to be required for our observed gene upregulation (Figure 2(b)). Consistent with the reduced response of cells to ABCG2 substrate anticancer drug (Table 1) after 2-DG and hypoxia treatment, ABCG2 efflux activity was found to be increased in all cell lines tested by the flow cytometry (Figure 3).

 Since MDR is usually mediated by overexpression of the MDR transporters, we intentionally investigated how ABCG2 regulation is affected by 2-DG and hypoxia in the ABCG2-overexpressing resistant S1M1-80 cell line. S1M1-80 has massive ABCG2 overexpression probably caused by a genetic translocation [10] and its escape from miR-519c repression [11] reported previously. ABCG2 protein expression was detected by Western blot analysis and cell surface staining. By both assays, 2-DG and hypoxia treatment were found to increase ABCG2 level (Figure 4), which is consistent with the elevated efflux activity (Figure 3) and reduced response to ABCG2 substrate anticancer drug (Table 1). Note that the massively overexpressed ABCG2 transcript level in S1M1-80 was not significantly affected by these treatments (Table 1); therefore, the observed functional effect may have been caused by modulation at the posttranscriptional and/or translational levels. This piece of data is important because it suggests that tumor microenvironment could coordinately regulate ABCG2 with preexisting MDR mechanisms to attain a higher resistance level. 

The ability of cancer cells to form colonies *in vitro* in an anchorage-independent manner is commonly used as a measure of their malignancy. After 2-DG and hypoxia treatment, both S1 and S1M1-80 cells were found to form more colonies in a soft agar colony formation assay (Figure 5). The upregulation of ABCG2 may play a specific role in this enhanced colony formation capability because genetic knockdown of ABCG2 was found to significantly reduce the number of colonies formed (Figure 5). Taken together, the three adverse growth conditions in the tumor microenvironment tested were found to upregulate ABCG2 to mediate multidrug resistance, with the possible enhancing effect on preexisting MDR mechanism, and to give rise to a more malignant phenotype. Novel clinical interventions may be devised to help circumvent this ABCG2-mediated resistance by modulating the tumor microenvironment. 

## Supplementary Material

Figure legend for Supplementary Figure 1: *ABCG2* promoter luciferase reporter gene assay showing that the promoter region harboring the HIF-1*α* response element is required for the activation of ABCG2 in HCT-116 human colon cancer cell line. Reporter activity in HCT-116 cells transiently transfected with the various *ABCG2* promoter constructs was measured with or without 24-h pretreatment with 2-DG (20 mM) or hypoxia. The mean reporter activity ± SD (firefly/renilla luciferase units *[*RLU*]*) from three independent experiments is shown.

## Figures and Tables

**Table 1:** Effect of a 16-h pretreatment of 2-DG (20 mM) or hypoxia on reducing the cytotoxic effect of ABCG2 substrate anticancer drug in HCT-116, S1 and S1M1-80.

Drug		IC_50_ ± SD	
	HCT-116	S1	S1M1-80
Mitoxantrone (*μ*M)			
Mitoxantrone alone	4.2 ± 1.2	0.15 ± 0.03	48 ± 2.3
with 2-DG	9.6 ± 1.5*	0.48 ± 0.04*	80 ± 4.1*
in Hypoxia	10.1 ± 1.3*	0.55 ± 0.04*	92 ± 7.9*
+FTC (5 *μ*M)	5.1 ± 1.1	0.13 ± 0.02	0.25 ± 0.10*

Cisplatin (*μ*g/mL)			
Cisplatin alone	8.3 ± 1.1	2.4 ± 0.9	3.3 ± 1.1
with 2-DG	6.5 ± 1.3	3.1 ± 1.0	4.9 ± 0.6
in Hypoxia	8.7 ± 1.6	3.5 ± 1.2	4.3 ± 1.3
+FTC (5 *μ*M)	9.2 ± 0.8	2.1 ± 0.8	4.6 ± 0.8

*P < 0.05, versus IC_50_ of the anticancer drug alone.

## Abbreviations

BCRP: Breast cancer resistance protein
FTC: Fumitremorgin C
HIF: Hypoxia inducible factor
MDR: Multidrug resistance
PhA: Pheophorbide A.
