# Supplementary figures and images for: Adverse Cell Culture Conditions Mimicking the Tumor Microenvironment Upregulate ABCG2 to Mediate Multidrug Resistance and a More Malignant Phenotype
Source: ISRN Oncol. 2012 Jun 14;2012:746025. doi: 10.5402/2012/746025 (PMC3384895; doi:10.5402/2012/746025)

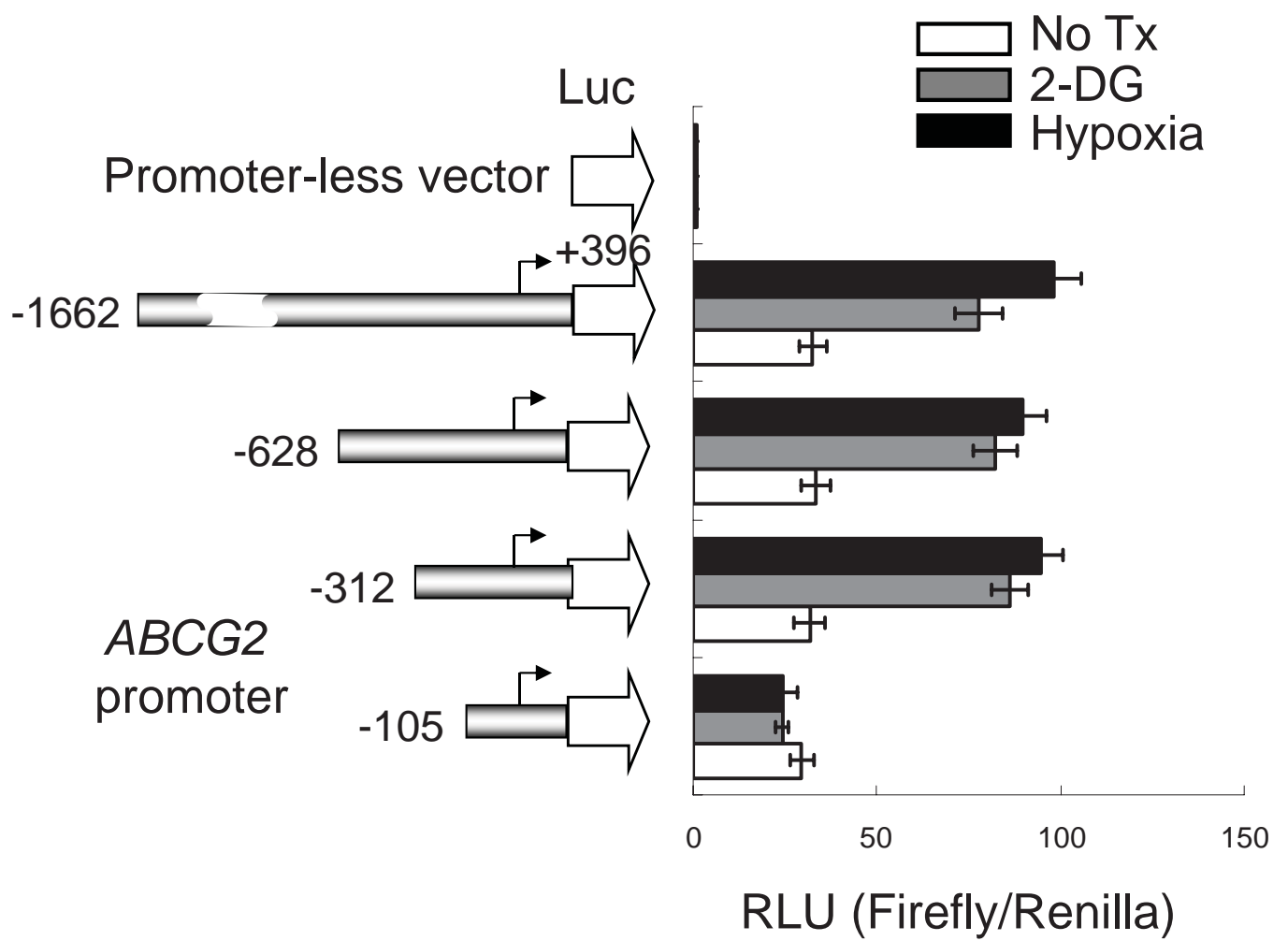

Supplement: Supplementary file 1 — Figure legend for Supplementary Figure 1: ABCG2 promoter luciferase reporter gene assay showing that the promoter region harboring the HIF-1α response element is required for the activation of ABCG2 in HCT-116 human colon cancer cell line. Reporter activity in HCT-116 cells transiently transfected with the various ABCG2 promoter constructs was measured with or without 24-h pretreatment with 2-DG (20 mM) or hypoxia. The mean reporter activity ± SD (firefly/renilla luciferase units [RLU]) from three independent experiments is shown. [file 746025.f1.pdf]
